# Apoptosis Exerts a Vital Role in the Treatment of Colitis-Associated Cancer by Herbal Medicine

**DOI:** 10.3389/fphar.2020.00438

**Published:** 2020-04-28

**Authors:** Ruimin Tian, Xianfeng Liu, Yanqin Luo, Shengnan Jiang, Hong Liu, Fengming You, Chuan Zheng, Jiasi Wu

**Affiliations:** ^1^College of Pharmacy, Chengdu University of Traditional Chinese Medicine, Chengdu, China; ^2^Department of Pharmacology, North Sichuan Medical College, Nanchong, China; ^3^Hospital of Chengdu University of Traditional Chinese Medicine, Chengdu, China; ^4^Innovative Institute of Chinese Medicine and Pharmacy, Chengdu University of Traditional Chinese Medicine, Chengdu, China

**Keywords:** apoptosis, herbal medicine, colitis-associated cancer, signaling pathways, inflammatory bowel disease

## Abstract

Colitis-associated cancer (CAC) is known as inflammatory bowel disease (IBD)-developed colorectal cancer, the pathogenesis of which involves the occurrence of apoptosis. Western drugs clinically applied to CAC are often single-targeted and exert many adverse reactions after long-term administration, so it is urgent to develop new drugs for the treatment of CAC. Herbal medicines commonly have multiple components with multiple targets, and most of them are low-toxicity. Some herbal medicines have been reported to ameliorate CAC through inducing apoptosis, but there is still a lack of systematic review. In this work, we reviewed articles published in S*ci Finder, Web of Science, PubMed, Google Scholar, CNKI*, and other databases in recent years by setting the keywords as apoptosis in combination with colitis-associated cancer. We summarized the herbal medicine extracts or their compounds that can prevent CAC by modulating apoptosis and analyzed the mechanism of action. The results show the following. (1) Herbal medicines regulate both the mitochondrial apoptosis pathway and death receptor apoptosis pathway. (2) Herbal medicines modulate the above two apoptotic pathways by affecting signal transductions of IL-6/STAT3, MAPK/NF-κ B, Oxidative stress, Non-canonical TGF-β1, WNT/β-catenin, and Cell cycle, thereby ameliorating CAC. We conclude that following. (1) Studies on the role of herbal medicine in regulating apoptosis through the Ras/Raf/ERK, WNT/β-catenin, and Cell cycle pathways have not yet been carried out in sufficient depth. (2) The active constituents of reported anti-CAC herbal medicine mainly include polyphenols, terpenoids, and saccharide. Also, we identified other herbal medicines with the constituents mentioned above as their main components, aiming to provide a reference for the clinical use of herbal medicine in the treatment of CAC. (3) New dosage forms can be utilized to elevate the targeting and reduce the toxicity of herbal medicine.

## Introduction

Colorectal cancer (CRC) is the third most common cause of malignancy incidence and death. In the United States alone, there were more than 140,000 newly diagnosed CRC patients and more than 50,000 deaths last year (2018) (Keum and Giovannucci, 2019). A recent epidemiological study announced that the early onset of CRC dominantly occurred among the white races and females (Glover et al., 2019*)*. Though the overall CRC rates have been reducing over recent years, evidence shows that there is a trend of increasing incidence among young people. Family medical history only accounts for about 20% of CRC cases, while environmental factors, obesity, smoking, alcohol abuse, and inflammatory bowel disease (IBD), in particular, are the main contributors (Serebriiskii et al., 2019*)*. Crohn’s disease (CD) and ulcerative colitis (UC) are the two defined IBD subtypes, and IBD-preceded CRC is known as colitis-associated cancer (CAC). Current data show that UC increases the cumulative risk of CAC by 18 to 20 percent, while CD increases the cumulative risk by 8 percent after 30 years. The exact overall increase in CAC prevalence in IBD patients depends on the severity and duration of the disease, the patient population analyzed, the availability of prophylactic colonoscopy in the general population, and the effectiveness of anti-inflammatory therapy (Al Bakir et al., 2018; Zhu et al., 2019*)*. The chronic inflammation-cancer relationship was first mentioned about one and a half centuries ago and had been confirmed by numerous clinical trials. Recent studies indicate that the most apparent correlation between long-term inflammation and tumor progression is observed in CAC, owing to the genetic change and epigenetic alteration elicited by inflammation (Romano et al., 2016; Hnatyszyn et al., 2019).

To date, the underlying molecular biological mechanisms of CRC are not fully understood, while there is a certain correlation between inflammation and cancer development, and the vital role of cytokines and various immune mediators in chronic tumorigenesis has been recognized (Qu et al., 2018). Multiple processes like tumor initiation and metastasis are involved in colitis-associated neoplasia. The pathogenesis of CAC is reported to be affected by multiple pathways, including TGF-β/SMAD, WNT/β-catenin, NOD/TLR, NLRP3 inflammasome, and the cell cycle as well as apoptosis, etc. (Nasuno et al., 2014; Choi et al., 2017; Cao and Xu, 2019), among which apoptosis is most focused on since the loss of adenomatous polyposis coli (APC) and TP53 mutations are crucial in IBD-CRC formation and IBD neoplasia initiation (Rogler, 2014).

In clinical settings, several approaches are taken in the treatment of CAC. For example, COX-2 inhibitor, aminosalicylates (5-ASA), and ursodesoxycholic acid have been clinically applied to target pathways like NF-κ B and oxidative stress (Foersch and Neurath, 2014). Meanwhile, a series of western medicines exert considerable ameliorative effect on CAC through modulating apoptotic pathways in animal experiments; the most typical are tauroursodeoxycholic acid (Kim et al., 2019), celecoxib (Setia et al., 2014), and simvastatin (Cho et al., 2008*)*. In addition, surgery, chemotherapeutic agents, and radiotherapy are employed for CAC patients in severe stage. Apart from western medicine mentioned above, there is a class of traditional herbal medicine, also known as ethnic drugs, that display great anti-CAC potential. At present, there are few reports on the treatment of CAC by herbal medicines through inducing apoptosis-associated signaling transductions, and there is a lack of relevant systematic review. In this article, we collect the details of the ameliorative effects of ethnic drugs on CAC, aiming to provide a reference for the future clinical use of ethnic dugs in CAC treatment.

## Apoptosis in Normal Intestinal Epithelia

Defined as a highly modulated physiological process of cell death, apoptosis is activated and regulated by a class of specific genes (Kaczanowski, 2016; Ismail et al., 2019). Upon activation, cells become rounded and retract from those nearby, after which apoptotic bodies are formed through the blebbing of dynamic plasma membrane. Meanwhile, nucleus condensation and the hydrolysis of nuclear DNA into fragments can be observed (Dhuriya et al., 2019). Unlike pyroptosis, which can trigger inflammatory response, the cell membrane structure is not damaged, and no contents are released during the whole apoptosis process (Zhang et al., 2019). Functionally, apoptosis maintains homeostasis and keeps the dynamic balance of cell numbers, and, as a defense mechanism, it eliminates abnormal cells (Norbury and Hickson, 2001). In brief, apoptosis can be activated by two separate pathways, namely the intrinsic and extrinsic pathways, referring to mitochondrial and death receptor pathways, respectively (Pfeffer and Singh, 2018).

Specifically, apoptosis affects intestinal physiology through maintaining normal colonic epithelia, the colonic crypt structure, and organ size (Keku et al., 2008; Ismail et al., 2019). In mammalian large intestine, epithelial cells are generated by the stem cells of the colonic crypts. The colonic crypt structure is affected by both apoptosis at the top of the crypt and cell proliferation at the bottom (Wang Y. et al., 2016; Kim et al., 2017; López-Posadas et al., 2017). The imbalance of apoptosis will result in the failure of colonic epithelial cell homeostasis and the cleaning-up of abnormal colonic epithelial cells, which eventually leads to colorectal cancer.

## Dysregulation of Apoptosis in CAC

As mentioned above, multiple genes have impacts on apoptosis, and the mutation or abnormal expressions of these genes may lead to apoptosis dysfunction. CAC is elicited from inﬂamed mucosa and progresses in the order of “inﬂammation-dysplasia-carcinoma” (Axelrad et al., 2016; Romano et al., 2016; Luo and Zhang, 2017). Prior to CAC, there is usually a long period of IBD. The typical characteristics of IBD are sustained mucosal inﬂammation with enhanced oxidative stress, promoted epithelium proliferation, and supported angiogenesis, which contributes to the initiation and progression of cancer (Romano et al., 2016; Luo and Zhang, 2017). It is reported that numerous cells or molecules, including immune cells, chemokines, stromal cells, epithelial cells, reactive oxygen species (ROS), and reactive nitrogen intermediates (RNI) and cytokines, participate in the modulation of the IBD microenvironment (Scarpa et al., 2014; Francescone et al., 2015).

Among them, ROS and RNI released by inflammatory cells directly damage colonic epithelium and, on the other hand, promote the genetic alterations driving carcinogenesis (Axelrad et al., 2016). The signaling transductions of JNK/MAPK and WNT/β-catenin are proved to be the key mechanism of colonic inflammation-tumor transformation (Kinugasa and Akagi, 2016). Besides, cytokines such as TNF-α, IL-1α/β, or IL-6 can trigger STAT3 signaling transduction and NF-κ B transcription, promoting tumor cell proliferation and survival as well as immune response (Klampfer, 2011; Kumari et al., 2016; Li et al., 2018). As a typical immune cell, the regulatory T cell is demonstrated to modulate the secretion of TNF-α or IL-6/11/22 so as to affect colonic cancer progression (Ju et al., 2017). On the other hand, TP53-mediated tumor cell apoptosis also exerts an important role in CAC pathogenesis. TP53 mutation is a key factor of apoptosis and is involved in IBD neoplasia initiation (Axelrad et al., 2016). Compared with p53, the dysfunction of which occurs early in CAC carcinogenesis, adenomatous polyposis coli (*APC*) gene mutation is elicited much later during the process of CAC tumorigenesis (Dyson and Rutter, 2012; Yaeger et al., 2016; Sanchez-Vega et al., 2018). It is reported that the loss of APC function will lead to insufficient β-catenin degradation, followed by the enhanced gene expression of survivin, and that survivin plays an important role in suppressing apoptosis through the inhibitory effect on caspase-3/7 and the release of cytochrome C. Moreover, some non-coding RNAs also play important roles in the apoptosis imbalance of CAC. miR-19a could promote CAC by regulating tumor necrosis factor alpha-induced protein 3-NF-κB feedback loops (Wang T. et al., 2016). It was found that miR-21-knockdown was associated with increased expression of PDCD4 gene and inhibition of NF-κ B activation as well as down-regulation of STAT3 and bcl-2 activation (Shi et al., 2016). In addition, it was reported that targeted deletion of mir-139-5p could activate MAPK, NF-κ B, and STAT3 signaling and decrease apoptosis and promote CAC (Zou et al., 2016). In summary, inhibition of apoptosis is a pivotal mechanism of CAC, and how to eﬀectively promote the apoptosis of colorectal cancer cells has clinical signiﬁcance for the treatment of CAC and may provide a feasible direction for the development of CAC-ameliorative drugs.

## Molecular Targets of Apoptosis Affected by Herbal Medicines in CAC

Since apoptosis inhibition is a crucial factor in CAC pathogenesis, inducing apoptosis of colorectal cancer cells can be regarded as an effective way to treat CAC. Apoptotic pathways include intrinsic and extrinsic apoptotic pathways, both of which are correlated with multiple other pathways, such as TGF-β/SMAD, WNT/β-catenin, NOD/TLR, and NLRP3 inflammasome, during the molecular nosogenesis of CAC. Thus, drugs that can induce apoptosis by acting on key targets of those pathways are promising candidates for the future treatment of CAC. Herbal medicine is a treasure house of medicine with multiple components and targets, and they have been believed to exert definite curative effects in the treatment of colorectal diseases for centuries. The following will detail the studies of herbal medicines and their components, as well as formulas for the treatment of CAC through modulating both intrinsic and extrinsic apoptotic pathways.

**Figure 1:**
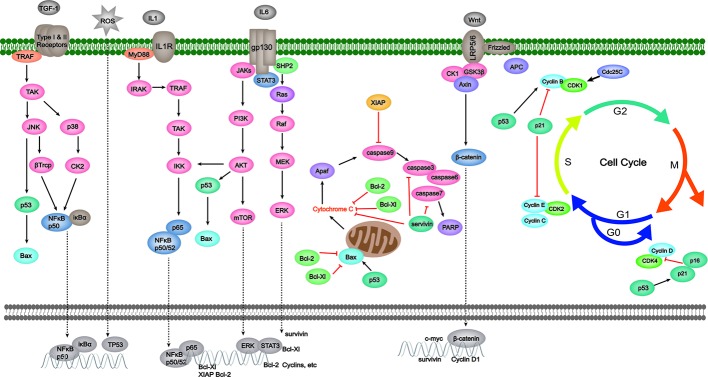
Effects of herbal medicine on the intrinsic pathway of apoptosis.

As depicted in **Figure 1**, the intrinsic pathway is also referred to as the mitochondrial-dependent apoptotic pathway and is regulated by the B-cell lymphoma 2 (Bcl-2) family of proteins (Zaman et al., 2014; Wang et al., 2019). The Bcl-2 family consists of not only pro-apoptotic proteins (Bax, Bak, etc.), but also anti-apoptotic proteins (Bcl-2, Bcl-Xl, etc.) (Siddiqui et al., 2015; Warren et al., 2019). In terms of mechanism, a variety of apoptotic stimuli mediate the over-expressions of BH3-only proteins, followed by the activations of both Bax and Bak in cytoplasm (Glab et al., 2017). They are then transferred to mitochondrial membrane, forming transmembrane pores and meanwhile reducing mitochondrial membrane potential (MCMP). After that, cytochrome C release is triggered because of the elevated permeability of mitochondrial membrane, resulting in apoptosome formation and the conversion from procaspase-9 to caspase-9 (Hassan et al., 2014; Zaman et al., 2014). This complex then activates several downstream effector caspases, such as caspase-3/6/7, and further induces DNA fragmentation and cell death (Anania et al., 2016; Tengku Din et al., 2018). Moreover, X-linked inhibitor of apoptosis protein (XIAP) and survivin, which are from the inhibitor of apoptosis proteins (IAP) family, can directly bind and inhibit key effector caspases such as caspase-3/7/9, thereby preventing apoptosis (Park et al., 2017).

Moreover, several other signaling pathways are also capable of regulating apoptosis through intrinsic means in the pathogenesis of CAC. Examples are as follows. (1) the activation of STAT3 induced by IL-6 is able to up-regulate the expression of several survival proteins like Bcl-2, Bcl-Xl, and survivin (Taniguchi and Karin, 2014; Siveen et al., 2014; Chonov et al., 2019). (2) NF-κ B, MAPK, and PI3K/AKT transductions are reported to initiate WNT/β-catenin signaling with or without APC, regulating apoptosis as well as neoplastic transformation(Mohammed et al., 2016), some of which also encode XIAP (Taniguchi and Karin, 2014; Romano et al., 2016; Evans et al., 2018). (3) The cyclin-dependent kinase inhibitor (CDKI) p21 is vital for p53-mediated G1/S boundary cell cycle arrest and cell senescence (Kim et al., 2017) (even although additional p53 target genes are also involved in the latter process).

**Figure 2:**
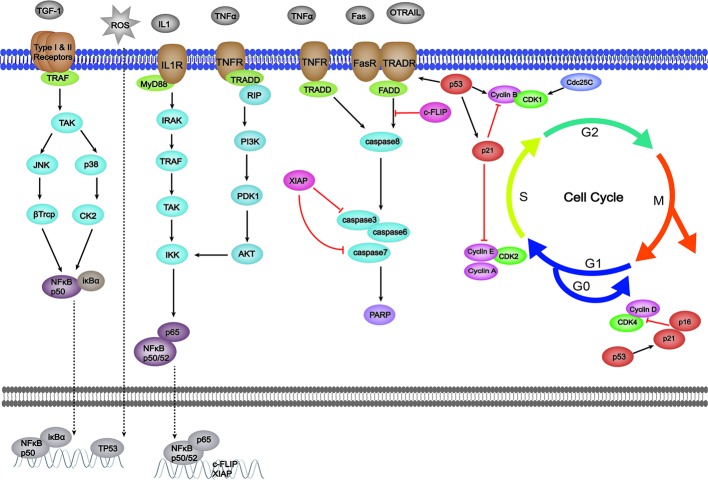
Effects of herbal medicine on the extrinsic pathway of apoptosis.

The extrinsic pathway is also known as the death receptor-mediated apoptotic pathway. Shown in **Figure 2**, this pathway is primarily activated by extracellular signals that are normally recognized by the proteins of the tumor necrosis factor receptor (TNFR) family (also termed death receptors), such as Fas (also known as CD95 or Apo1), TRAIL-R, and TNFR (Ntuli, 2012; Sharma et al., 2019; Ian and Chaudhry, 2019). The extracellular signals mainly contain Fas ligand (Fas-L), TNF-related apoptosis-inducing ligand (TRAIL), and tumor necrosis factor (TNF), and the gene expression of Fas can be promoted by p53 (Goldar et al., 2015; Ray et al., 2016). The binding of ligands and their specific receptors then recruits death signal adaptor proteins, such as Fas-associated death domain (FADD) and TNF receptor-associated death domain (TRADD), to the death receptors (Zaman et al., 2014; Liu et al., 2017). After that, the death-inducing signaling complex (DISC) is formed by the oligomerized receptors and recruited adaptor proteins (Qiao and Benjamin, 2009). DISC can bind to procaspase-8 and produce active caspase-8, thereby promoting the activation of caspase-3/6/7 and leading to apoptotic events. In addition, the activation of caspase-8 is also a link between extrinsic and intrinsic pathways through the activation of BID (Green and Llambi, 2015). Cellular FLICE-like inhibitory protein (c-FLIP) is an inhibitor of DISC (Zaman et al., 2014). Not only inhibiting the intrinsic pathway, XIAP also plays a role in extrinsic pathway modulation through potently inhibiting executioner caspase-3/7 (Obexer and Ausserlechner, 2014). Furthermore, NF-κ B hyper-activation also can be observed in CAC for its contributions to the up-regulation of XIAP, c-FLIP, and p53 mutation (Grivennikov, 2013). The specific effects of herbal medicine on those signaling pathways are as follows.

### Monomers From Herbal Medicine

**Figure 3:**
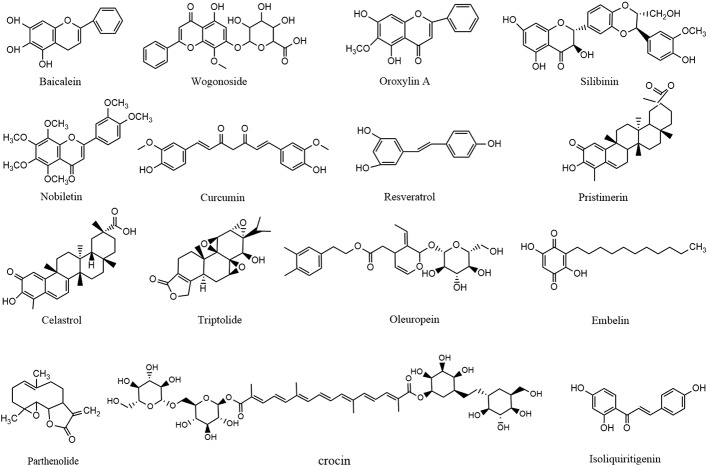
Molecular structures of reported anti-CAC compounds from herbal medicine.

Of monomers, polyphenols are the most widely reported treatment for CAC (shown in **Figure 3**). A study conducted by Kim et al. demonstrated that administration of baicalein (1–10 mg/kg for 14 weeks) from *Scutellaria baicalensis* Georgi downregulated expressions of pro-caspase-3/8 so as to induce HCT-116 apoptosis through extrinsic means in a mouse model of colitis-driven colon cancer (Kim D. H. et al., 2013). Also from *Scutellaria baicalensis* Georgi, wogonoside (100 mg/kg/d for 15 weeks) was reported to increase the survival rate of AOM/DSS-induced CAC mice by decreasing tumor number, tumor size, average tumor load, and occurrence of large-sized adenomas through the reduction of NF-κ B p65, p-p65, PI3K, p-Akt, cyclin D1, and survivin levels, as well as cytokine secretion in tumor tissue (Sun et al., 2016). Analogously, Yang et al. indicated that oroxylin A (50-200 mg/kg/d for 100 days), an active ingredient in *Scutellaria baicalensis* Georgi, also exerted a CAC-ameliorative property. In their study, 5-aminosalicylic acid (5-ASA) was set as a positive drug. The underlying molecular mechanism included its inhibitory effect on STAT phosphorylation as well as the expressions of Bcl-2 and cyclin D. Besides, the Bax level was elevated after oroxylin A administration, suggesting that oroxylin A induced apoptosis through modulating the IL-6/STAT3 pathway in ADM/DSS-elicited mouse colitis-associated carcinogenesis (Yang et al., 2013). Furthermore, Zheng et al. demonstrated that silibinin (750 mg/kg for 10 weeks, dissolved in 0.5% carboxymethyl cellulose) from *Silybum marianum* (L.) Gaertn also ameliorated CAC by affecting STAT3/IL6R signaling transduction (Zheng et al., 2018). Similarly, in 2017, a study reported that nobiletin from dried tangerine peel and its colonic metabolites could suppress colitis-associated colon carcinogenesis. In the study, AOM/DSS induced CRC mice were fed an AIN93G diet supplemented with nobiletin (0.05 wt% in diet for 20 weeks), after which the incidence and multiplicity of colonic tumors were reduced, and there were also reductions in expression of iNOS and protein levels of cyclin D, CDK6, CDK4, and CDK2 and increased levels of p27 and p53 (Wu et al., 2017). Extracted from another famous herbal medicine named *Curcuma longa* L., curcumin (25 mg/kg/day for 62 days) promoted the accumulation of cells in G0/G1 phase and subsequently induced tumor cell apoptosis by regulating targets that involved WNT/β-catenin like cyclin D1 in AOM/DSS-challenged mice (Marjaneh et al., 2018). In addition, resveratrol (0.03 wt% in diet for 9 weeks) from white hellebore was demonstrated to reduce macroscopic lesions, dysplasia, and inflammation in colon of CAC model mice. The effect was attributed to downregulation of the levels of iNOS, COX-2, TNF-α, and p53 (Cui et al., 2010). Moreover, isoliquiritigenin (20–500 μg/ml in diet for 12 weeks) extracted from licorice could significantly reduce the incidence of tumor in colon of AOM/DSS-induced CAC mice, which involved the decrease of iNOS, COX-2, and CD206 levels (Feng, 2017)

Terpenoids have also been repeatedly reported to be used to treat CAC. In 2018, researchers found that pristimerin (0.0001~0.0005 wt% in diet for 10 weeks) isolated from *Tripterygium wilfordii* Hook F could inhibit cell proliferation and enhance apoptosis through regulating the cell cycle progression of colon cancer cells, because it was active in modulating targets like cyclin D1, CDC25A, p27, p21, caspase-3/7/8/9, and cleaved-PARP (Park et al., 2018). Besides, Celastrol, another main constituent in *Tripterygium wilfordii* Hook F, is also considered as a potential candidate for CAC therapy. Also, in a mouse model of CAC established by AOM/DSS treatment, celastrol (2 mg/kg/d for 14 weeks) was observed to significantly decrease the levels of oncogenic proteins such as p53 and β-catenin. In addition, the levels of TNF-α, IL-1β, IL-6, COX-2, and iNOS were also reduced through the inhibitory effect of celastrol on the NF-κ B signaling pathway (Lin et al., 2015). Triptolide (0.1,0.3,1m g/kg/d for 20 weeks), also extracted from *Tripterygium wilfordii* Hook F, was reported to suppress the development of colitis and colon cancer through inducing cell apoptosis and regulating the IL-6/JAK/STAT pathway by inhibiting cyclinD1/CDK4 expression and STAT3/IL6R/JAK1 levels, respectively (Wang et al., 2009).

In addition to polyphenols and terpenoids, oleuropein (50–200 mg/kg/d for 8 weeks) from *Olea europaea* L was confirmed to be a promising protective agent ameliorating CAC to do its ability to prevent colon inflammation, epithelial damage, and tumor formation in colon. Further investigations also utilized 5-ASA as positive drug and verified that the mechanism involves oleuropein’s positive effect on Bax and negative effect on p65, β-catenin, STAT3, and Akt expressions (Ginger et al., 2016). Similarity, embelin (50 mg/kg/d for 85days) from *Embelia ribes* Burm. F. is a well-known XIAP inhibitor and is capable of modulating p-STATS and IL-6 to decrease in the BrdUrd incorporation in dysplastic areas so that CAC progression is limited (Dai et al., 2014). In another study, parthenolide (2–4 mg/kg/d for 68 days) from *Tanacetum parthenium* significantly decreased the number of nodular, polypoid, and caterpillar−like tumors in the middle and distal colon of mice and meanwhile alleviated the severity of inflammation in the colons. Those effects are exerted through suppressing the expression of IκBα and p65, down-regulating the Bcl−2 and Bcl−Xl levels, and enhancing caspase-3 expression (Kim et al., 2015). Besides, crocin (0.005,0.01,0.02 wt% in diet for 15 weeks) from *Crocus sativus* L. was also proved to reverse the over-incidence of severe inflammation with mucosal ulcers and high-grade dysplastic crypts. The underlying mechanism is associated with its regulatory effect on NF-κ B, COX-2, iNOS, TNF-α, IL-1β, and IL-6 (Kawabata et al., 2012).

### Herbal Medicine Extracts

**Figure 4:**
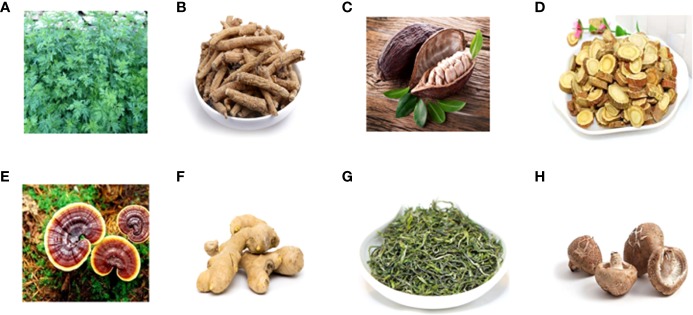
Reported anti-CAC herbal medicines. **(A)** Artemisia princeps Pampanini cv, **(B)** American ginseng, **(C)** Cocoa, **(D)** Licorice, **(E)** Mushroom Ganoderma lucidum, **(F)** Ginger, **(G)** Camellia sinensis L.O. Kuntze, **(H)** Lentinula edodes.

Chung et al. conducted research to evaluate the effect of standardized ethanol extract from the aerial parts of *Artemisia princeps Pampanini* cv. *Sajabal via* (EAPP) on AOM/DSS-induced CAC, as depicted in **Figure 4**. The results showed EAPP (25 mg/kg/day, three times a week for 9 weeks) could inhibit the pro-inflammatory and pro-proliferative activities that were mediated by NF-κ B and trigger apoptotic response. Briefly, EAPP decreased p65 expression and protein levels of NF-κ B-dependent pro-survival genes, such as Bcl-2, XIAP, cFLIP, and survivin, as well as inducing caspase-3/8/9 activations (Chung et al., 2015). The hexane fraction of *American ginseng* (11.9mg/kg/d for 35/50 days) also suppressed CAC progression because it was capable of reducing macroscopic lesions and microscopic colon adenomas and meanwhile blocking inflammation and cancer markers. Mechanically, AG enhanced the p53 level and reversed the over-expressions of iNOS and COX-2 so as to induce apoptosis occurrence in inflammatory cells, CD4þ/CD25 effector T cells, and lymphocytes (Poudyal et al., 2012). In addition, another study found that American ginseng could significantly downregulate the expression of cytokines (IL-1α, IL-1β, IL-6, G-CSF, and GM-CSF) and restore the balance of the metabolomics and intestinal flora, especially increasing the expression of Firmicutes while downregulating Bacteroidales and Verrucomicrobia (Wang C. Z. et al., 2016). In the same CAC model, cocoa (5–10% in diet for 62 days) displayed considerable CAC-ameliorative properties by elevating the Bax and caspase-3 levels while diminishing levels of Bcl-Xl and pro-inflammatory cytokines (TNF-α, IL-1β, IL-6, and IL-17), resulting in the reverse of shortening colon length and weight loss in AOM/DSS-challenged mice (Saadatdoust et al., 2015). As well as the signaling pathways mentioned above, the NF-κ B/JAK2/STAT3 signaling pathway was focused on in another study examining whether flavonoids extracted from Licorice prevented CAC development. Licorice flavonoids (0-100 mg/kg for 10 weeks) did affect apoptotic targets (Bax and Bcl-Xl), proliferation-associated targets (proliferating cell nuclear antigen (PCNA), p53, p21, and cyclinD1), and inflammation-associated targets (p-JAK2, p-STAT3, IKKα/β, and p-IκBα), showing considerable potential value of clinical use against CAC (Huo et al., 2016). In another CAC mouse model established by a food-borne carcinogen (2-Amino-1-methyl-6phenylimidazol [4, 5-b] pyridine [PhIP]) plus DSS, Trierpene extract isolated from *Mushroom Ganoderma* lucidum (GLT) exerted significant ameliorative effect. The tumor incidence and multiplicity were suppressed after GLT administration (0–500 mg/kg for 17 weeks), and the mechanism was revealed to be correlated with the inhibition of cyclin D1 and COX-2 expression in colon tissue (Sliva et al., 2012). Furthermore, a study showed that oral administration of ginger-derived nanoparticles (GDNPs) (0.3 mg/mouse/day for 19 weeks) could significantly reduce the incidence and growth of CAC in AOM/DSS-induced mice. The underlying molecular mechanisms were related to the down-regulation of proliferation marker cyclin D1 and pro-inflammatory cytokines such as IL-6, IL-1β, and TNF-α (Zhang M. et al., 2016). HLiu et al. reported that tea polysaccharides (TPS) (0–200mg/kg for 13weeks) from *Camellia sinensis* L.O. Kuntze inhibited AOM/DSS-induced development of CAC cancer, promoted the apoptosis ratio in a mouse model, and suppressed cell proliferation in CT26 cells *via* arresting the cell cycle through modulating the expression of cyclin D1, MMP-2, and MMP-9 (Liu L. Q. et al., 2018). Similarly, tea polyphenols, the main components of tea, exerted good effect on the treatment of CAC in mice. It was observed that tea polyphenols (0.1% in water for 42 days) inhibited the formation of tumor through down-regulating the expression of COX-2, TNF-α, IL-6, β-catenin, and C-myc and up-regulating the expression of IL-4 and IL-10 (Mo et al., 2017). As a kind of polysaccharide exerted from Lentinula edodes, Lentinan (0-20mg/kg for 7-21 days) could also produced anti-cancer effects in an AOM/DSS-induced CAC mice model by inhibiting the TLR4/NF-κ B signaling pathway (Perse and Cerar, 2012; Liu Y. et al., 2018).

### Chinese Medicinal Formulae

**Figure 5:**
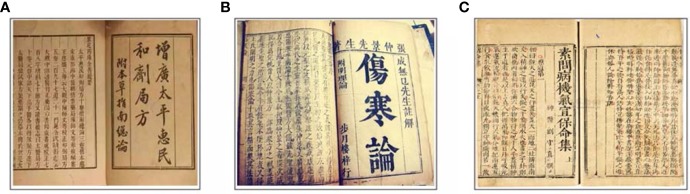
Anti-CAC formulas documented in ancient books. **(A)** The prescriptions of the Bureau of Taiping People’s Welfare Pharmacy describing Shenling Baizhu San. **(B)** ‘Shang Han Lun’ documenting Huangqin Decoction. **(C)** ‘Plain Questions - anthology on proper therapy for Qi disorder and pathogenesis to save life’ describing Shaoyao Decoction.

Shenling Baizhu San, presented in **Figure 5**, is also a well-known Chinese medicine formula, which is comprised of ten commonly used herbs (as shown in **Table 2**) and has been used for the treatment of gastrointestinal disorders for centuries. In the study of Lin et al., SBS (7.28 g/kg for 15 weeks) administration was able to significantly down-regulate the levels of neoplastic markers such as PCNA, β-catenin, and p53 as well as TGF-β1 and Wnt5a in a mouse model of CAC established by AOM/DSS treatment (Lin et al., 2015). Huangqin Decoction (HQD), composed of *Scutellaria baicalensis* Georgi, *Paeonia lactiflora* Pall, *Glycyrrhiza uralensis* Fisch, and *Ziziphus jujuba* Mill, is a famous formula from the Shang Han Lun that has been widely used in the ﬁght against gastrointestinal symptoms (Zhang et al., 2010; Chen et al., 2015). HQD (9.1g/kg/day for 16 weeks) was observed to increase the survival rate, prevent the shortening of colon length, and reduce the number of tumors, tumor size, and tumor load of AOM/DSS-induced CAC mice after oral gavage. Further research indicated that HQD could reduce the levels of TNF-α, IL-1β, IL-6, CSF-1, MCP-1, and COX-2, which suggested that HQD might improve CAC through its anti-oxidative and anti-inflammation properties (Chen et al., 2016). In addition, another canonical Chinese medicine prescription named ShaoYao decoction (SYD) was reported to have a mitigating effect on CAC. SYD includes nine herbs: *Radix Paeoniae* Alba, *Radix Angelicae* Sinensis, *Rhizoma Coptidis* L, *Semen Arecae*, *Radix Aucklandiae*, *Radix Et Rhizoma* Glycyrrhizae, *Radix Et Rhizoma* Rhei, *Radix Scutellariae*, and *Cortex Cinnamomi*. After receiving SYD (7.12 g/kg for 15 weeks), increased survival rate and reduced incidence and multiplicity of colonic neoplasms were detected in AOM/DSS-induced CAC mice. The authors concluded that the alleviating effects of SYD on CAC were through inhibiting the expression levels of PCNA, β-catenin, COX-2, and p53 in colon tissue. Moreover, the levels of IL-1β, IL-6, TNF-α, and NF-κ B were also down-regulated by SYD, suggesting that SYD might ameliorate CAC by suppressing NF-κ B activation (Lin et al., 2014).

## Herbal Medicines Based on the Above Active Components

**Figure 6:**
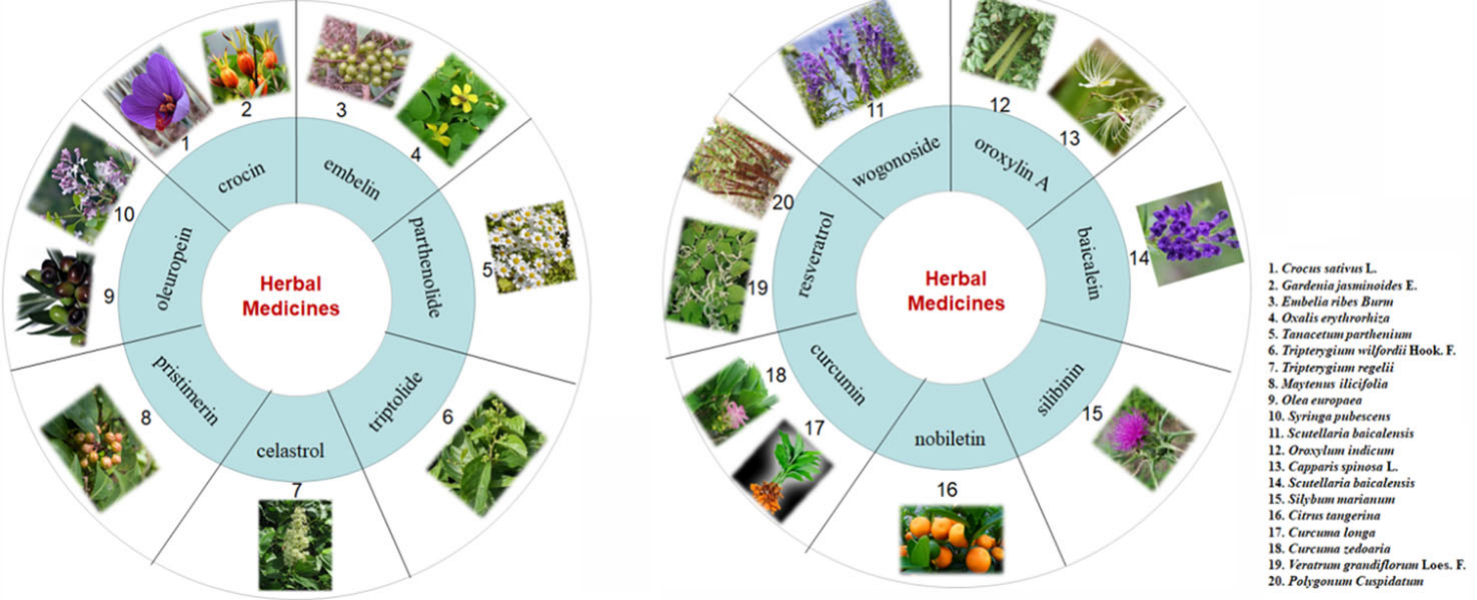
Other sources of reported monomers with anti-CAC properties.

The active components mentioned above for the treatment of CAC are also widely distributed in many medicinal plants (**Figure 6**). Crocin is a water-soluble carotenoid that is found in the stigma of saffron (*Crocus sativus* L.) and the fruit of gardenia (*Gardenia jasminoides* Ellis) (Pham et al., 2000). Embelin is a benzoquinone that is purified from the fruits of *Embelia ribes* Burm. F and is also found in other medicinal plants, such as *Oxalis acetosella* L. and *Lysimachia punctata* L. (Lu H. et al., 2016). Parthenolide, which is extracted from the shoots of feverfew (*Tanacetum parthenium*), is a kind of sesquiterpene lactone (Ghantous et al., 2013). Oleuropein is one of the most abundant active components contained in the leaves of the olive tree (*Olea europaea* L.), and it can be isolated from many other medicinal plants like *Syringa pubescens* Turcz, *Ligustrum lucidum* Ait, and *Chionanthus virginicus* L. (Hassen et al., 2015). It is of interest that most of the active constituents are either polyphenols or terpenoids. Among them, wogonoside, oroxylin A, baicalein, silibinin, nobiletin, curcumin, and resveratrol are polyphenols. Of note, wogonoside, oroxylin A, and baicalein are all active constituents of *Scutellaria baicalensis* Georgi., a Chinese traditional medicine that has been widely used for thousands of years. It is worth mentioning that oroxylin A is also found in *Oroxylum indicum* (L.) Kurz. (Lu L. et al., 2016). Silibinin is mainly derived from the seeds of milk thistle (*Silybum marianum* (L.) Gaertn.). In addition, nobiletin is mainly present in the peels of tangerine (*Citrus tangerina*), sweet orange (*Citrus sinensis* (L.) Osbeck), and bitter orange (*Citrus aurantium* L) (Li et al., 2014), curcumin has a two-century history and can be extracted from *Curcuma longa* L., *Curcuma zedoaria* (Christm.) Rosc., and *Curcuma aromatica* Salisb. (Aggarwal et al., 2007). Pure resveratrol was first isolated from the roots of white hellebore (*Veratrum grandiflorum* O. Loes) in 1940, and it has also been found to exist in the roots of *Rheum rhaponticum* L. (Huang et al., 2019). The active ingredient in licorice, which plays an anti-CAC role, is another polyphenol (Huo et al., 2016). In addition, *Artemisia princeps Pampanini* cv. Sajabal and cocoa are also rich in polyphenols (Ju et al., 2012).

Apart from polyphenols, terpenoids are the second-largest group of active constituents for CAC treatment, among which the typical ones are triptolide, celastrol, and pristimerin. Celastrol is a quinone methide triterpenoid first extracted from the root bark of the Chinese medicine ‘Thunder of God Vine’ (also known as *Tripterygium wilfordii* Hook F) in 1936 (Yang et al., 2006; Hou et al., 2019). Pristimerin (a species of quinone methide triterpenoid) and triptolide (a species of diterpenoid triepoxide) are also capable of being isolated from *Tripterygium wilfordii* Hook F (Kim H. J. et al., 2013; Hou et al., 2019; Zhang C. et al., 2017). It is surprising that these three components are all active constituents of *Tripterygium wilfordii* Hook F and that they are also abundant in the *Celastraceae* and *Hippocrateaceae* families (Kim H. J. et al., 2013; Venkatesha et al., 2016). Moreover, Ganoderma triterpenoids also exert considerable ameliorative effects on CAC (Sliva et al., 2012). The details of the sources of the main ingredients are as follows.

## Adverse Effects

Overall, no serious adverse effects have been reported by the studies conducted in herbal medicine (Wu J. et al., 2019; Payab et al., 2020). There was reportedly a low risk of adverse events associated with the uses of *American ginseng, Licorice, Ginger*, and *Lentinula edodes* in maintaining well-being, decreasing nausea or vomiting in early-stage pregnant women, ameliorating postoperative sore throat, and preventing nodal metastasis in breast cancer patients, respectively (Arring et al., 2018; Stanisiere et al., 2018; Kuriyama and Maeda, 2019). However, heavy metal contents in *Coca*, such as nickel and aluminum, displayed potential adverse non-carcinogenic health effects in consumers (Salama, 2018). In one study, adverse effects like gastrointestinal, skin, and subcutaneous tissue disorders were observed after the consumption of *Artemisia princeps* Pampanini cv (Vitalone et al., 2012). As for *Mushroom Ganoderma* lucidum, most participants tolerate it well, and only three episodes of toxicity were recorded until 2016; two patients had nausea, and one experienced insomnia (Jin et al., 2016). Moreover, the mutagenicity and acute and subchronic toxicity of *Camellia sinensis* L.O. Kuntze was examined in a rat model, and the results indicated that the level of no observed adverse effects was 4.0 g/kg bw/day (Li et al., 2011).

## Future Prospects

Conventional therapies for CAC include tauroursodeoxycholic acid (Kim et al., 2019), celecoxib (Setia et al., 2014), and simvastatin et (Cho et al., 2008). However, the high risk of side effects seriously influences the quality of life of patients. Furthermore, long-term use may lead to drug resistance and reduce efficacy. In recent years, more and more attention has been paid to the applications of herbal medicine to CAC treatment. Compared to synthetic drugs, herbal medicine exerts characteristics of multiple-targeting and correlatively low adverse effects. Over the past decades, numerous studies have been conducted to make advances in anti-CAC investigations of herbal medicine. On the basis of CAC pathogenesis, this work aimed to evaluate the efficacy, underlying mechanisms, and safety of reported herbal medicine in CAC treatment. To date, more than 20 kinds of herbal medicine are confirmed to have CAC-ameliorative properties, and the underlying mechanisms through apoptosis modulation are shown in **Figures 1**, **2** and **Table 1**. We found that apoptosis imbalance in CAC is closely related to anti-cancer gene mutations (TP53, APC, etc.) that are caused by oxidative stress occurrence and the abnormal signaling transductions of multiple pathways (such as MAPK, NF-κ B, and JAK/STAT) as well as the elevated expressions of anti-apoptotic proteins (such as Bcl-2/Xl, XIAP, and c-FLIP). At present, herbal medicines have been proved to be effective in preventing CAC progression by affecting apoptosis in both intrinsic and extrinsic ways, as well as regulating the MAPK, NF-κ B, and JAK/STAT pathways. Apparently, the majority of research efforts have mainly concentrated on the mechanisms involved in colonic inflammatory response and colon cancer apoptosis and metastasis, while modulation on the cell cycle arrest, Ras/Raf/ERK, and WNT/β-catenin pathways, respectively accounting for tumor cell proliferation, invasion, and premalignant lesion, have not been thoroughly studied. In addition, one study, through mitochondrial proteomics, identified an important differential protein named six-transmembrane epithelial antigen of prostate 4 (STEAP4), which was highly expressed and promoted mitochondrial iron accumulation and oxidative stress, thus promoting the occurrence of CAC. The study further found that the hypoxia/HIF–2 alpha/STEAP4/mitochondrial iron/mitochondrial ROS axis promoted colitis and colon cancer development (Xue et al., 2017). As yet, there have been no studies on the improvement of CAC by herbal medicine through modulating STEAP4 or related signaling pathways. Further studies are needed. On the other hand, the dose-effect relationship is a criterion for judging how well a drug works, and toxicity is supposed to be taken into consideration first when evaluating efficacy. We notice that, the dose range of monomer, extracts, and formula is 0–1000 μM/1–750 mg/kg (cell/animal), 0–2 mg/mL/0–500 mg/mL (cell/animal), and 0–16 μg/mL/7.12–9.1 g/kg (cell/animal), respectively. Generally, herbal medicines exert low toxicity and have no serious side effects even with a long period of use; the typical side effects are slight skin or gastrointestinal disorders like skin rash and nausea. This suggests that herbal medicines have a relatively wide range of safety (Payab et al., 2020).

**Table 1:** Effects of the monomers, extracts, or formulas of herbal medicines on apoptosis-related pathway molecules.

Herbal medicine	Cell	Dose	Treatment time	Animal	Dose	Treatment time	Pathway	Related targets	Reference
Baicalein	HCT-116	25, 50, 100 μM	24 h	Male ICR mice (five weeks old)	1, 5, 10 mg/kg/d	14 w	NF-κB, extrinsic pathway	Pro-caspase-3/-8/-9, PARP, pIκBα, p50, p65, iNOS, MMP-2 ↓ cleaved PARP, PPARγ ↑	Kim D. H. et al., 2013
Wogonoside	HCT116, HT29, THP-1 cells	50, 100, 150 μM	24 h	C57BL/6 mice (6-8 weeks old)	100 mg/kg	15 w	P13K/AKt, NF-κB pathway	P13K, p-AKt, IKKα, IκBα, NF-κB, p65, p-p65, IL-1β, IL-6, TNF-α, Cyclin D1, survivin ↓	Sun et al., 2016
Oroxylin A	HCT-116	25, 50, 100 μM	1 h	Male and female C57BL/6 mice (6-8 weeks old)	50, 100, 200 mg/kg/d	100 d	NF-κB, IL-6/STAT3 pathway	IL-6, IL-1β, p-STAT3, STAT3↓	Yang et al., 2013
Silibinin	IMCE and HCT-116	50, 100, 200, 400, 800 μM	72 h	Female C57BL/6J mice (6 weeks old)	750 mg/kg/d	10 w	IL-6/STAT3 pathway	IL-6, IL-1β, TNF-α, p-STAT3↓	Zheng et al., 2018
Nobiletin and its metabolites	RAW 264.7 cells, HCT116 cells	0.5, 1, 2 μg/ml	24 h	Male CD-1 mice	500 ppm nobiletin in diets	20 w	Nrf2 pathway, Cell cycle pathway	iNOS, cyclinE, cyclinD, CDK6, CDK4, CDK2 ↓ Nrf2, HO-1, NQO1, p21, p27, p53↑	Wu et al., 2017
Phytosomal curcumin	CT-26	0-1000 μM	24/48/72 h	Female C57/6 mice (8 weeks old)	25 mg/kg/d	62 d	WNT /β-catenin pathway	β-catenin,cyclin D1 ↓	Marjaneh et al., 2018
Resveratrol				Male and female C57BL/6 mice (8–12 weeks old)	300 ppm resveratrol in diets	9 w	Oxidative stress, NF-κB pathway	iNOS, COX-2, TNF-α, p53↓	Cui et al., 2010
Isoliquiritigenin				Male BALB/c mice (6 weeks old)	20,100,500 μg/ml isoliquiritige in diets	12 w	Oxidative stress	iNOS, COX-2, CD206↓	Feng, 2017
Pristimerin				Female BALB/c mice (5 weeks old)	1-5 ppm Pristimerin diets	10 w	NF-κ B, AKT/FOXO3a pathway, extrinsic/intrinsic pathway, cell cycle	P-AKT, p-FOXO3a, TNF-α, IL-6, Q-iNOS, COX-2, IκB-α, PCNA, cyclinD1, CDC25A, Bcl-2, NF-κB, Bcl-Xl, p65↓ p21, p27, cleaved caspase-3, -7, -8, and -9, cleaved PARP ↑	Park et al., 2018
Celastrol	HCT116, HT-29 cells	0-40 μM	24,48h	Male C57BL/6 mice (6-8 weeks old), male BALB/c-nu mice (5 weeks old)	2 mg/kg/d 1, 2 mg/kg	14 w 18 d	NF-κB pathway, p53-related pathway, EMT-related pathway	NF-κBp65, TNF-α, IL-6, IL-1β, COX-2, iNOS, p53, p-p53, N-cadherin, Vimentin, Snail, β-catenin ↓ E-cadherin ↑	Lin et al., 2016
Triptolide	SW480 cells Caco2 cells	10, 30, 100, 300 nM	24,48,72 h	Male ICR mice	0.1, 0.3, 1 mg/kg/d	20 w	JAK/STAT3 pathway, Cell cycle pathway	IL-6R, IL-6, JAK1, STAT3, Rac1, Cyclin D1, CDK 4 ↓	Wang et al., 2009
Crocin				Male ICR Mice (4 weeks old)	50, 100, 200 ppm crocin in diets	15/4 w	NF-κB signaling pathway	NF-κ B, COX-2, iNOS, TNF-α, IL-1β, IL-6 ↓ Nrf2 ↑	Kawabata et al., 2012
Oleuropein				Female C57BL/6 mice	50, 100 mg/kg	8 w	NF-κB pathway, WNT/β-catenin pathway, IL-6/STAT 3 pathway, P53-related pathway, P13K/AKt pathway	p65, NF-κ B, TNF-α, β-catenin, Cox-2, IL-6, STAT3, AKt, IFN-γ, IL-17A↓ Bax ↑	Ginger et al., 2016
Embelin	HCT116	20 μmol/L	1-24 h	Male C57BL/6 mice (6-8weeks)	50 mg/kg	85 d	IL-6/STAT3 pathway	IL-6, p-STAT3, IL-1β, IL-17a, IL-23 ↓ p-SHP2 ↑	Dai et al., 2014
Parthenolide				Female Balb/C mice (6 weeks old)	2, 4 mg/kg	68 d	NF-κB pathway	IĸBα, NF-ĸB-p65, Bcl-2, Bcl-Xl ↓ Caspase-3 ↑	Kim et al., 2015
Extract of *Artemisia princeps Pampanini* cv. *Sajabal* (EAPP)	HT-29 HT-116	60, 120, 180 μg/ml	6 h	Male ICR mice	25 mg/kg/d three times a week	9 w	NF-κB extrinsic/intrinsic pathway	P65, survivin, cFLIP, cIAP, XIAP, Bcl-2, Bcl-Xl, Mcl-1, TNF-α,P- IL-1β↓ Q-PARP-1↑	Chung et al., 2015
American Ginseng (Hexane fraction)	ANA-1 murine macrophage, TK6 lymphoblastoid cells, CD4^+^/CD25^-^ effector T cells	260 μg/ml, 0-1000 μg/ml, 0-300 μg/ml	24 h	Mice	11.9 mg/kg/d	35/50 d	Oxidative stress pathway	iNOS, COX-2 ↓	Poudyal et al., 2012
Cocoa				Female BALB/c mice	5%, 10% cocoa in diets	62 d	NF-κB/ IL-6/STAT3, extrinsic pathway	Bcl-Xl, IL-6, CD68^+^, PCNA, IL-17, IL-1β, TNF-α, NF-κB, p-STAT3^Y705^↓ Bax, caspase-3↑	Saadatdoust et al., 2015
Licorice flavoids (LFs)				Female C57BL/6 mice	50, 100 mg/kg/d	10 w	NF-κB/IL-6/Jak2/Stat3, p53 pathway	iNOS, Cox-2, IL-1β, IL-6, TNF-α, PCNA, NF-κB, IKKα/β,p-IκBp-Jak2, p-Stat3, Bcl-2, CyclinD1↓ Bax, p-P53, P21↑	Huo et al., 2016
Ganoderma lucidum triterpene extract				Male ICR mice (5weeks old)	0, 100, 500 mg/kg (three times per week)	17 w	NF-κB, cell cycle pathway	CyclinD1, COX-2, CYP1A2, CYP3A4 ↓	Sliva et al., 2012
Nanoparticles derived from edible ginger	RAW264.7 cells, Caco-2BBE, Colon-26 cells	0-100 μg/ml	24h	Female C57BL/6 or FVB/NJ mice (6-8 weeks old)	0.3 mg/mouse	19 w	Cell cycle pathway	TNF-α, IL-6, IL-1β, CyclinD1↓ IL-10, IL-22 ↑	Zhang M. et al., 2016
Tea polysaccharide (TPS)	CT-26 cells	20-320 μg/ml	48h	BALB/c mice	0-200 mg/kg	13 w	Cell cycle pathway	CyclinD1, MMP-2, MMP-9↓	Liu L. Q. et al., 2018
Tea polyphenols				Male BALB/c mice (4 weeks old)	0.1% in water	42 days	WNT/β-catenin pathway	COX-2, TNF-α, IL-6, β-catenin, C-myc ↓ IL-4, IL-10↑	Mo et al., 2017
Lentinan	RAW264.7 cells	0.5, 1, 2 mg/ml	24h	Female C57BL/6 mice, BALB/c mice	5, 10, 20 mg/kg, 5, 10, 20 mg/kg, 20 mg/kg	7 or 20 d, 7 d 21 d	TLR4/NF-κB pathway	MyD88, IRAK4, TRAF6, IKBKB, NF-κB-p65, NF-κB, IL-13, CD30L ↓	Liu Y. et al., 2018
Huangqin Decoction				Male C57BL/6 mice (8-week old)	9.1 g/kg	16 w	Oxidative stress pathway	TNF-α, IL-1β, IL-6, CSF-1, MCP-1, COX-2, MPO, MDA,8-oxoguanine, nitrotyrosine ↓	Chen et al., 2016
ShaoYao decoction (SYD)				Male C57BL/6J mice (6and 8 weeks old)	7.12 g/kg, twice a day	15 w	NF-κB pathway	β-catenin, COX-2, p53, p65, PCNA, N-cadherin, fibronectin, vimentin, Snail, IL-1β, IL-6, TNF-α↓ E-cadherin↑	Lin et al., 2014
Shenling Baizhu San	SW480, HCT116. Cells	0-16 μg/ml	12/24h	Male C57BL/6 J mice	7.28 g/kg (twice a day)	15 w	TGF-β1, WNT/β-catenin pathway	TGF-β1, Wnt5a, β-catenin, PCNA, p53, N-cadherin, vimentin, Fibronectin, Snail ↓ E-cadherin, Axin, Dvl2, GSK-3β↑	Lin et al., 2015

Emerging evidence has shown that the efficacy of herbal medicine can be improved by promoting bioavailability by chemical structure modification and dosage form transformation. It is of note that herbal medicines, especially their monomer contents, have low oral bioavailability, in some cases no more than 1% (Wu J. S. et al., 2019). To date, some new, natural, and low-toxicity drug delivery systems have been developed in experimental studies, such as ginger-derived nanoparticles, that could target the inflamed bowel mucosa and show a promising direction for the prevention and treatment of IBD and CAC (Zhang M. et al., 2016).

In terms of ingredient categories, the active constituents of reported anti-CAC herbal medicines mainly include polyphenols, terpenoids, and saccharide. We therefore collected information on other herbal medicines that have the ingredients mentioned above as their main constituents (displayed in **Table 2**), even though there is no evidence indicating their CAC-ameliorative activation so far. For example, *Zanthoxylum bungeanum* Max (ZBM), also known as Szechuan pepper, has been used in the fight against gastrointestinal disorders for centuries. It is cultivated over a wide geographical range and has substantial production, much higher than that of other medicinal herbs (Li et al., 2016). The essential oil and pericarp of ZBM have been demonstrated to have a positive impact on experimental colitis *via* the regulation of the NF-κ B/PPAR-γ and TLR4-related signaling pathways, respectively (Zhang Z. et al., 2016; Zhang Z. et al., 2017), suggesting its great potential to be a CAC-ameliorative candidate. Similarly, the traditional Chinese medicine ‘Qing Dai (*Indigo naturalis*)’ also showed therapeutic effects on ulcerative colitis and could be a promising anti-CAC drug (Suzuki et al., 2013). Furthermore, it is worth mentioning that the active constituents responsible for the anti-CAC properties of herbal medicines like ginger, American ginseng, and Shaoyao decoction have not been clearly identified and still need further investigation.

**Table 2:** Herbal medicines based on the above active components.

Monomers	Medicinal plants	Reference
Crocin	*Crocus sativus* L*., Gardenia jasminoides* Ellis.	Pham et al., 2000
Embelin	*Embelia ribes* Burm. F.*,Oxalis acetosella* L*.,Lysimachia punctata* L.	Lu H. et al., 2016
Parthenolide	*Tanacetum parthenium*.	Ghantous et al., 2013
Oleuropein	*Olea europaea L.,Syringa pubescens* Turcz.*,Syringa reticulata* (Blume) Hara., *Syringa dilatata,Syringa oblata* Lindl*,Osmanthus cymosus,Ligustrum vulgare,Ligustrum lucidum* Ait*., Fraxinus excelsior* Linn*., Fraxinus ornus Linn.*, *Osmanthus fragrans* (Thunb.) Lour.*, Fraxinus americana* Linn.*, Chionanthus virginicus L., Fraxinus angustifolia, Phillyrea angustifolia L.,Phillyrea latifolia L*.	Hassen et al., 2015
Wogonoside	*Scutellaria baicalensis* Georgi.	Sun et al., 2016
Oroxylin A	*Scutellaria baicalensis* Georgi.*,Stachys geobombycis* C. Y. Wu.*,Oroxylum indicum* (L.) Kurz*, Capparis spinosa* L*,Eucommia ulmoides* Oliver.	Lu L. et al., 2016
Baicalein	*Scutellaria baicalensis* Georgi.	Kim D. H. et al., 2013
Silibinin	*Silybum marianum* (L.) Gaertn.	Zheng et al., 2018
Nobiletin	*Citrus tangerina,Citrus sinensis* (L.) Osbeck*,Citrus aurantium* L.	Li et al., 2014
Curcumin	*Curcuma longa L.,Curcuma mangga,Curcuma zedoaria* (Christm.) Rosc*.,Costus speciosus,Curcuma xanthorrhiza,Curcuma aromatica* Salisb*.,Curcuma phaeocaulis,Etlingera elatior, Zingiber cassumunar*.	Aggarwal et al., 2007
Resveratrol	*Veratrum grandiflorum* (Maxim.) Loes. F.*,Cassia* sp.*,Polygonum Cuspidatum,Arachis hypogaea* Linn*., Eucalyptus robusta* Smith*,Vitis vinifera* L.*,Morus alba L.,Picea* sp.*,Vaccinum* sp.*,c,Artocarpus* sp.*,Rheum rhaponticum* L*.,Reynoutria japonica* Houtt.*,Gnetum montanum* Markgr*., Bauhinia purpurea* L*., Pinus sylvestris L., Veratrum* sp.	Huang et al., 2019
Celastrol	*Tripterygium wilfordii* Hook F*,Tripterygium hypoglaucum* (Levl.) Hutch,*.Tripterygium regelii* Sprague et Takeda.	Kim H. J. et al., 2013; Venkatesha et al., 2016
Pristimerin	*Maytenus ilicifolia,Celastrus orbiculatus* Thunb*.Celastrus hypoleucus* (Oliv.) Warb.ex Loes.*, Salacia oliveriana,Maytenus Heterophylla,Maytenus senegalensis (Lam.) Exell.,Triterpygium wilfordii* Hook F.	Kim H. J. et al., 2013
Triptolide	*Tripterygium wilfordii* Hook F.*, Celastrus orbiculatus* Thunb*., Tripterygium hypoglaucum* (Levl.) Hutch.	Zhang C. et al., 2017

Last but not least, the existing studies on the herbal medicine-CAC relationship remain at the experimental level; data from clinical trials are limited. The effectiveness and the relevant underlying molecular mechanism through which herbal medicines play a role as anti-CAC drugs are still unclear. Our results confirm the effectiveness of herbal medicine such as *Artemisia princeps* Pampanini cv, *American ginseng*, and *Licorice* in the treatment for CAC through apoptosis modulation. However, there is still a desperate need to determine better anti-CAC drug candidates with more potent effects and lower levels of side effects. Furthermore, the pathogenesis of CAC has not been fully elucidated, and the apoptosis-related pathways regulated by herbal medicine are not fully explored. It will be meaningful to continue to track progress on the impact of herbal medicine on CAC.

To sum up, herbal medicines are promising drugs with multiple regulatory targets, especially in apoptosis-related pathways, while more knowledge is still needed before they meet clinical requirements and can be further applied to the prevention and therapy of CAC. In addition, more efforts are needed to promote the bioavailability of herbal medicines as well as their monomer contents so as to reach a balance between therapeutic efficacy and toxicological safety.

## Author Contributions

RT and XL wrote the draft. YL searched the literature. JW and FY drew the figures. SJ prepared the tables. HL checked literature and data. CZ and JW supervised the work.

## Funding

This work was supported by the National Key Research and Development Program of China [2017YFC1703904], the National Natural Science Foundation of China [81773974, 81774284 and 81803994], and the International Cooperation Project of Sichuan Science and Technology Department [2019YFH0152]. Science and Technology Developmental Foundation of Chengdu University of TCM [No.XSGG2019019].

## Conflict of Interest

The authors declare that the research was conducted in the absence of any commercial or financial relationships that could be construed as a potential conflict of interest.

## Abbreviations

AG, American ginseng; AOM, azoxymethane; APC, adenomatous polyposis coli; ASA, aminosalicylates; Bcl-2, B-cell lymphoma-2; Bcl-Xl, B-cell lymphoma-Extra Large; CAC, Colitis-associated cancer; CD, Crohn’s disease; CDC25A, cell division cycle 25A; CDK2, cyclin-dependent kinase 2; CDK4, cyclin-dependent kinase 4; CDK6, cyclin-dependent kinase 6; CDKI, cyclin-dependent kinase inhibitor; c-FLIP, cellular FLICE like inhibitory protein; COX-2, Cyclooxygenase-2; CRC, Colorectal cancer; DISC, death-inducing signaling complex; DSS, dextran sodium sulfate; EAPP, extract from the aerial parts of Artemisia princeps Pampanini cv.; FADD, Fas-associated death domain; Fas, factor associated suicide; Fas-L, Fas ligand; GLT, Trierpene extract isolated from Mushroom Ganoderma lucidum; GDNPs, ginger-derived nanoparticles; HQD, Huangqin Decoction; IAP, inhibitor of apoptosis proteins; IBD, inflammatory bowel disease;IL-1α, interleukin-1α; IL-1β, interleukin-1β; IL-6, interleukin-6; IL-17, interleukin-17; iNOS, inducible nitric oxide synthase; JAK: janus kinase; MAPK, mitogen-activation protein kinase; MCMP, mitochondrial membrane potential; NF-κB, nuclear factor-κB; NLRP3, nucleotide oligomerization domain (NOD)-like receptor protein-3; NOD/TLR, nucleotide binding oligomerization domain/toll like receptors; PCNA, proliferating cell nuclear antigen; Ras, rat sarcoma; Raf, RAF proto-oncogene serine/threonine protein kinase; ERK, extracellular regulated protein kinases; RNI, reactive nitrogen intermediates; ROS, reactive oxygen species; STAT, signal transducers and activators of transcription; STAT3, signal transduction and activation of transcription 3; SYD, ShaoYao Decoction; TNF, tumor necrosis factor; TNF-α, tumor necrosis factor-α; TGF-β1, transforming growth factor-β1; TNFR, tumor necrosis factor receptor; TPS, Tea polysaccharides; TRADD, TNF receptor-associated death domain; TRAIL, TNF-related apoptosis-inducing ligand; UC, ulcerative colitis; XIAP, X-linked inhibitor of apoptosis protein.
